# Supplementary material for: Improving deep neural network generalization and robustness to background bias via layer-wise relevance propagation optimization
Source: Nat Commun. 2024 Jan 4;15:291. doi: 10.1038/s41467-023-44371-z (PMC10767127; doi:10.1038/s41467-023-44371-z)
Supplement: Supplementary file 3 — Reporting Summary [file 41467_2023_44371_MOESM3_ESM.pdf]

nature portfolio

Corresponding author(s): Pedro Ricardo Ariel Salvador Bassi and Andrea Cavalli

Last updated by author(s): Nov 26, 2023

Reporting Summary

Nature Portfolio wishes to improve the reproducibility of the work that we publish. This form provides structure for consistency and transparency in reporting. For further information on Nature Portfolio policies, see our Editorial Policies and the Editorial Policy Checklist.

Statistics

For all statistical analyses, confirm that the following items are present in the figure legend, table legend, main text, or Methods section.

n/a	Confirmed	
☐	☒	The exact sample size (n) for each experimental group/condition, given as a discrete number and unit of measurement
☐	☒	A statement on whether measurements were taken from distinct samples or whether the same sample was measured repeatedly
☒	☐	The statistical test(s) used AND whether they are one- or two-sided<br>*Only common tests should be described solely by name; describe more complex techniques in the Methods section.*
☒	☐	A description of all covariates tested
☒	☐	A description of any assumptions or corrections, such as tests of normality and adjustment for multiple comparisons
☐	☒	A full description of the statistical parameters including central tendency (e.g. means) or other basic estimates (e.g. regression coefficient) AND variation (e.g. standard deviation) or associated estimates of uncertainty (e.g. confidence intervals)
☒	☐	For null hypothesis testing, the test statistic (e.g. F, t, r) with confidence intervals, effect sizes, degrees of freedom and P value noted<br>*Give P values as exact values whenever suitable.*
☐	☒	For Bayesian analysis, information on the choice of priors and Markov chain Monte Carlo settings
☒	☐	For hierarchical and complex designs, identification of the appropriate level for tests and full reporting of outcomes
☒	☐	Estimates of effect sizes (e.g. Cohen's d, Pearson's r), indicating how they were calculated

Our web collection on statistics for biologists contains articles on many of the points above.

Software and code

Policy information about availability of computer code

Data collection	The data was collected from open sources and downloaded manually, except for the TB Portals data, which was downloaded with Python (3.9) and pandas (1.4.2).
Data analysis	The data was analyzed utilizing custom code, described in the manuscript. Novel algorithms are available at https://github.com/PedroRASB/ISNet. The new code is mainly based on PyTorch (version 1.11.0), PyTorch Lightning (1.6.3), and Python (3.9). All benchmark models were also based on the same packages. Additional supporting and required Python packages were CUDA (11.3.1), cuDNN (8.2.0), NumPy (1.21.5), TorchVision (0.12.0), Matplotlib (3.5.1), opencv-python (4.5.2), SciPy (1.7.3), scikit-image (0.19.2), scikit-learn (0.23.2), pandas (1.4.2), dill (0.3.4), Jupyter Notebook (4.7.1), and PyTorch GradCAM (1.4.6). Bayesian analyses were performed with PyMC3 (3.1). The DeepMAC network used to create segmentation ground-truth masks for the Stanford Dogs dataset employed TensorFlow (2.10.0).

For manuscripts utilizing custom algorithms or software that are central to the research but not yet described in published literature, software must be made available to editors and reviewers. We strongly encourage code deposition in a community repository (e.g. GitHub). See the Nature Portfolio guidelines for submitting code & software for further information.

Data

Policy information about availability of data

All manuscripts must include a data availability statement. This statement should provide the following information, where applicable:

- Accession codes, unique identifiers, or web links for publicly available datasets
- A description of any restrictions on data availability
- For clinical datasets or third party data, please ensure that the statement adheres to our policy

Source data are provided with this paper. The X-ray data from healthy and/or pneumonia positive subjects used in this study are available in the Montgomery and Shenzen databases, http://archive.nlm.nih.gov/repos/chestImages.php; in ChestX-ray14, https://paperswithcode.com/dataset/chestx-ray14; and in CheXpert, https://stanfordmlgroup.github.io/competitions/chexpert/. The COVID-19 radiography data used in this study are available in The BrixIA COVID-19 project, https://brixia.github.io/; and in the BIMCV-COVID19+ database, https://bimcv.cipf.es/bimcv-projects/bimcv-covid19/. The tuberculosis-positive X-rays used in this study are available in NIAID TB Portals, https://tbportals.niaid.nih.gov/download-data. The Images for facial attribute estimation (along with their segmentation masks) used in this study are available in the Large-scale CelebFaces Attributes (CelebA) Dataset, https://mmlab.ie.cuhk.edu.hk/projects/CelebA.html. The MIMIC-CXR-JPG database (v2.0.0) used in this study is available at https://physionet.org/content/mimic-cxr-jpg/2.0.0/. The Stanford Dogs dataset used in this study is available at http://vision.stanford.edu/aditya86/ImageNetDogs/. X-rays with lesions marked by the radiologist and the corresponding ISNet Layer-wise Relevance Propagation heatmaps (Supplementary Data 1), generated in this study, have been deposited in https://doi.org/10.6084/m9.figshare.24243895.v2. All data supporting the findings described in this manuscript are available in the article and in the Supplementary Information and from the corresponding author upon request.

Human research participants

Policy information about studies involving human research participants and Sex and Gender in Research.

Reporting on sex and gender	n/a
Population characteristics	n/a
Recruitment	n/a
Ethics oversight	n/a

Note that full information on the approval of the study protocol must also be provided in the manuscript.

Field-specific reporting

Please select the one below that is the best fit for your research. If you are not sure, read the appropriate sections before making your selection.

☒ Life sciences ☐ Behavioural & social sciences ☐ Ecological, evolutionary & environmental sciences

For a reference copy of the document with all sections, see nature.com/documents/nr-reporting-summary-flat.pdf

Life sciences study design

All studies must disclose on these points even when the disclosure is negative.

Sample size	The study considers multiple datasets and the various corresponding classification tasks (COVID-19 classification, tuberculosis classification, classification of multiple lung conditions, facial attribute estimation, and dog breed classification). Thus, the applications assess the proposed neural network's robustness to background bias in diverse conditions (biomedical data or natural images, with varying task difficulties and dataset sizes), and better delimit the model's use-case scenario. In this study, we employed the largest high-quality and open COVID-19 and tuberculosis X-ray datasets that we were able to find. X-rays with other findings (pneumonia and healthy) were gathered from substantial open repositories, in similar or larger quantities, better balancing the databases. The CheXPert and MIMIC chest X-ray datasets (used in Supplementary Note 7) were chosen because they are among the largest and most popular open databases for lung disease classification. Facial attribute estimation considered a subset of the popular CelebA database, comprising a large number of samples (30,000). Finally, the Stanford Dogs subset is the smallest dataset in this study (501 samples). Thus, it assesses the network's capabilities with smaller databases, considering a challenging fine-grained classification task. The smallest dataset's size was still sufficient for the observation of considerable differences in the performance metrics of the multiple neural networks.
Data exclusions	Unlike other classes, the COVID-19 databases had a very small quantity of pediatric patients. Thus, we excluded pediatric patients from other databases in all experiments involving COVID-19 X-rays, to avoid biasing the neural networks.
Replication	We employed open databases and thoroughly described the methods used in their analysis. We made custom code available.
Randomization	The X-ray experiments employed diverse open databases for training/hold-out validation, and for out-of-distribution testing. The training and hold-out validation datasets were randomly splitted in all experiments. For facial attribute estimation and dog breed classification, we considered the standard test datasets published by the database authors, who created them by randomly splitting the data into a train and a

	test partition.
Blinding	Before the completion of the task, the radiologist that manually analyzed X-rays had no access to the neural networks, their outputs, or their heatmaps.

Reporting for specific materials, systems and methods

We require information from authors about some types of materials, experimental systems and methods used in many studies. Here, indicate whether each material, system or method listed is relevant to your study. If you are not sure if a list item applies to your research, read the appropriate section before selecting a response.

Materials & experimental systems

n/a	Involved in the study
☒	☐ Antibodies
☒	☐ Eukaryotic cell lines
☒	☐ Palaeontology and archaeology
☒	☐ Animals and other organisms
☒	☐ Clinical data
☒	☐ Dual use research of concern

Methods

n/a	Involved in the study
☒	☐ ChIP-seq
☒	☐ Flow cytometry
☒	☐ MRI-based neuroimaging
