# Supplementary material for: Improving deep neural network generalization and robustness to background bias via layer-wise relevance propagation optimization
Source: Nat Commun. 2024 Jan 4;15:291. doi: 10.1038/s41467-023-44371-z (PMC10767127; doi:10.1038/s41467-023-44371-z)
Supplement: Supplementary file 5 — Code and Software Policy [file 41467_2023_44371_MOESM5_ESM.pdf]

nature research

Corresponding author(s): Pedro R. A. S. Bassi and Andrea Cavalli

Code and Software Submission Checklist

Prior to submitting your work to Nature Research, we strongly recommend that you ask at least one colleague who is unfamiliar with your software to install the tool(s), follow the instructions, and provide feedback. This process will help ensure that reviewers will also be able to run your software.

You must submit all required content as a single zip file prior to peer review or provide a link where editors and reviewers can access all required content.

Required content

- [x] Compiled standalone software and/or source code
- [x] A small (simulated or real) dataset to demo the software/code

A README file that includes:

1. System requirements
   - [x] All software dependencies and operating systems (including version numbers)
   - [x] Versions the software has been tested on
   - [x] Any required non-standard hardware
2. Installation guide
   - [x] Instructions
   - [x] Typical install time on a "normal" desktop computer
3. Demo
   - [x] Instructions to run on data
   - [x] Expected output
   - [x] Expected run time for demo on a "normal" desktop computer
4. Instructions for use
   - [x] How to run the software on your data
   - [x] (OPTIONAL) Reproduction instructions

     We encourage you to include instructions for reproducing all the quantitative results in the manuscript.

Additional information

Describe your software's license for use. We strongly recommend using a license approved by the Open Source Initiative.

BSD 3-Clause "New" or "Revised" License

Provide a link to the code in an open source repository (when available).

https://github.com/PedroRASB/ISNet

Your manuscript should include a complete, detailed description of the code's functionality (i.e. pseudocode).

Please indicate where this is found:

- [ ] Main text
- [x] Methods section
- [ ] Elsewhere (specify):

Examples of well-structured software packages

1. https://github.com/neurodata-papers/MGC
2. https://github.com/neurodata-papers/LOL
3. https://www.nature.com/nbt/journal/v34/n6/abs/nbt.3569.html#supplementary-information
4. https://www.nature.com/nature/journal/v548/n7669/full/nature23463.html#extended-data
   https://github.com/yasharhezaveh/Ensai
5. https://www.nature.com/nbt/journal/v34/n11/full/nbt.3685.html#supplementary-information
   https://github.com/IFIproteomics/LFQbench
